# Classification of Non-Small Cell Lung Cancer Using Significance Analysis of Microarray-Gene Set Reduction Algorithm

**DOI:** 10.1155/2016/2491671

**Published:** 2016-06-30

**Authors:** Lei Zhang, Linlin Wang, Bochuan Du, Tianjiao Wang, Pu Tian, Suyan Tian

**Affiliations:** ^1^School of Life Science, Jilin University, 2699 Qianjin Street, Changchun, Jilin 130012, China; ^2^Department of Neurology, The Second Hospital of Jilin University, 218 Ziqiang Street, Changchun, Jilin 130041, China; ^3^Division of Clinical Epidemiology, The First Hospital of Jilin University, 71 Xinmin Street, Changchun, Jilin 130021, China; ^4^School of Mathematics, Jilin University, 2699 Qianjin Street, Changchun, Jilin 130012, China

## Abstract

Among non-small cell lung cancer (NSCLC), adenocarcinoma (AC), and squamous cell carcinoma (SCC) are two major histology subtypes, accounting for roughly 40% and 30% of all lung cancer cases, respectively. Since AC and SCC differ in their cell of origin, location within the lung, and growth pattern, they are considered as distinct diseases. Gene expression signatures have been demonstrated to be an effective tool for distinguishing AC and SCC. Gene set analysis is regarded as irrelevant to the identification of gene expression signatures. Nevertheless, we found that one specific gene set analysis method, significance analysis of microarray-gene set reduction (SAMGSR), can be adopted directly to select relevant features and to construct gene expression signatures. In this study, we applied SAMGSR to a NSCLC gene expression dataset. When compared with several novel feature selection algorithms, for example, LASSO, SAMGSR has equivalent or better performance in terms of predictive ability and model parsimony. Therefore, SAMGSR is a feature selection algorithm, indeed. Additionally, we applied SAMGSR to AC and SCC subtypes separately to discriminate their respective stages, that is, stage II versus stage I. Few overlaps between these two resulting gene signatures illustrate that AC and SCC are technically distinct diseases. Therefore, stratified analyses on subtypes are recommended when diagnostic or prognostic signatures of these two NSCLC subtypes are constructed.

## 1. Introduction

Lung cancer (LC) is one of the leading causes of death worldwide, with approximately 85% of LC cases being non-small cell lung cancer (NSCLC) [1]. NSCLC can be further divided into three major subtypes, among which adenocarcinoma (AC) and squamous cell carcinoma (SCC) account for roughly 40% and 30% of all LC cases, respectively [2]. Since AC and SCC differ in their cell of origin, location within the lung, and growth pattern, they are considered as distinct diseases [3].

Gene expression signatures have been demonstrated to be capable of distinguishing AC and SCC apart [3–6]. When building up such a signature, a feature selection algorithm is usually implemented to deal with the high dimensionality of gene expression profiles [7]. Among a variety of feature selection algorithms, many incorporate coexpressed/coregulated information contained within pathways to facilitate the selection of relevant features. Applications of those algorithms to real-world microarray data have shown that accounting for such information can always improve predictive power and biological interpretation of a classifier [8–10].

Gene set analysis is regarded as irrelevant to the identification of individual gene expression signatures because it considers a whole gene set's concordant association with a phenotype. However, it is found that some gene set analysis algorithms can be utilized directly for selecting relevant genes and obtaining a diagnostic or prognostic gene signature [11]. Significance analysis of microarray- gene set reduction (SAMGSR) [12] is one of such algorithms. It extends significance analysis of microarray-gene set method (SAMGS) [13], which accumulates the squared SAM statistics over all genes inside a gene set to determine this gene set's significance, with one reduction step. SAMGSR aims to further downsize the selected gene sets into their respective core subsets. This reduction step essentially carries out feature selection.

In previous work [11], we applied SAMGS to a real-world microarray dataset and identified the gene signatures discriminating multiple sclerosis (MS) patients from their controls. Although the resulting signatures perform well, the application of SAMGS to MS data encounters one big disadvantage; MS is one disease under less investigation compared to cancers and thus its associated gene sets are far from being comprehensively annotated by those major databases such as KEGG [14] and GO [15]. This drawback might conceal the actual capability of feature selection held by SAMGSR.

While microarray technology remains popular, RNA-sequencing (RNA-seq) has evolved quickly and become a competitive choice to profile genes' expression values [16]. With the aid of a recently proposed R Bioconductor function called Voom [17], the application of statistical methods originally proposed for microarray data to RNA-seq read count data becomes feasible. It facilitates integrated analysis of data from these two technologies. For example, previous studies have justified that KRT5 plays a critical role in the segmentation of AC and SCC using both microarray and RNA-seq platforms [5].

NSCLC is a multistage progression process resulting from genetic sequences mutations; thus genes associated with NSCLC patients at histology stage I and with those at stage II might differ potentially. Nevertheless, none of recent efforts by the industrial methodology for process verification in research (IMPROVER), diagnostic signature challenge (DSC) [18], and ours [4, 5, 19] had achieved successful stage I versus stage II segregations for AC or SCC.

In this study, we aim to address several issues by applying SAMGSR to NSCLC microarray and RNA-seq data. First, taking advantage of the fact that the gene sets associated with NSCLC are well annotated in major canonical databases, SAMGSR will be further explored on its use as a feature selection method. Second, by incorporating extra pathway knowledge contained in lung cancer relevant gene sets, we intend to obtain gene signatures capable of discriminating different stages within each subtype apart. Finally, we aim to test the generalization of resulting gene signatures on a larger cohort even collected from a different platform.

## 2. Materials and Methods

### 2.1. Experimental Data

Microarray data are available in the Gene Expression Omnibus (GEO) repository (Accession number: GSE50081) and RNA-seq data are in The Cancer Genome Atlas (https://tcga-data.nci.nih.gov/tcga/). Since both datasets were utilized by us previously for constructing prognostic gene signatures of NSCLC, we refer to that work [20] for the details and skip the descriptions on them here.

### 2.2. Preprocessing Procedures

For the RNA-seq data, counts-per-million (CPM) values were calculated and log_2_ transformed by Voom function [17] in R limma package [21]. For the microarray data, expression values were obtained using the GCRMA algorithm [22], and quantile normalization was carried out and then the expression values were log_2_ transformed. These two datasets were preprocessed and normalized separately.

There are 16,363 unique genes annotated by both hgu133 plus2 and RNA-seq platforms. Given the differences between these two datasets, for example, different platforms used and personal characteristics, we performed an integrative correlation (IC) [23] analysis to exclude genes with inconsistent expression patterns across studies. Then, SAMGSR analysis was carried out using the 7,286 genes that passed the IC filtering.

### 2.3. Statistical Methods

#### 2.3.1. SAMGSR

As an extension of SAMGS, SAMGSR reduces the number of genes contained in the pathways selected by SAMGS up to 90% [12]. It consists of two major steps. First, SAMGS is used to select relevant pathways. Subsequently, each selected pathway is refined to a concise subset. In SAMGS step, the following statistic is defined for gene set *j*:

(1) $$\begin{array}{c} {\text{SAMGS}}_{j}=\overset{\left|j\right|}{\underset{i=1}{\sum }}d_{i}^{2},\;d_{i}=\frac{\left({\overset{-}{x}}_{d}\left(i\right)-{\overset{-}{x}}_{c}\left(i\right)\right)}{\left(s\left(i\right)+s_{0}\right)}, \end{array}$$

where *d*_*i*_ is SAM statistic [24] and ${\overset{-}{x}}_{d}\left(i\right)$ and ${\overset{-}{x}}_{c}\left(i\right)$ are the sample averages of gene *i* for the diseased group and the control group, respectively. *s*(*i*) is a pooled standard deviation and estimated by pooling samples over two groups and *s*_0_ is a small positive constant used to offset the small variability in microarray measurements. The size of gene set *j*, that is, the number of genes contained in gene set *j*, is denoted as |*j*|. Technically, SAMGS statistic is the *L*_2_ norm of SAM statistics for all genes within the gene set.

For a significant gene set identified by SAMGS, where its statistical significance is determined using permutation tests by permuting phenotype-labels for several hundred times, SAMGSR gradually partitions the entire set *S* into two subsets: the reduced subset *R*_*k*_ including the first *k* genes and its complement set ${\overset{-}{R}}_{k}$ for *k* = 1,…, |*j*| after the genes in the gene set are ordered decreasingly based on the absolute values of their SAM statistics. Let *c*_*k*_ be the *p* value of SAMGS statistic for ${\overset{-}{R}}_{k}$; the final size of *R*_*k*_ corresponds to the smallest *k* when *c*_*k*_ is larger than a threshold *c*.

#### 2.3.2. Implementation of SAMGSR for Feature Selection

As mentioned above, SAMGSR extends SAMGS by adding an extra step of reducing the selected gene sets to their respective core subsets. We note that this additional reduction step is a process of feature selection in nature. There are two cutoffs in SAMGSR. One is the significance level *α* in SAMGS, which determines the number of gene sets selected by SAMGS. The other is *c*, which determines the size of reduced core subsets. Both of them are considered as tuning parameters in a supervised learning process and determine jointly the sparseness of the final model.

To determine the optimal values of those two parameters, we conducted a grid search by varying their values over two sets of values (i.e., 0.01 and then 0.05 to 0.3 with an increment of 0.05 for *c* and 0.01, 0.05, 0.1, 0.15, and 0.2 for the significance level in SAMGS) and using 5-fold cross-validations. The set of *c* and *α* achieving the best performance in terms of discriminative ability on the cross-validated data was chosen. Then, with these two parameters being fixed at their optimal values, SAMGSR was applied again on the training data set to select potentially relevant genes. Furthermore, a support vector machine (SVM) [25] model using those genes selected by SAMGSR was fitted to calculate the performance statistics.

### 2.4. Integrative Correlation Analysis

The integrative correlation (IC) analysis was used to filter out the genes exhibiting incoherent behavior across studies. As discussed in its original paper [23] and by us [26], those incoherent genes are highly likely to be noises. Here, we give a brief introduction to IC.

For the specific study *s*, let *x*_*g*_ represent the expression profile for a gene *g*; then *ρ*_*p*_^*s*^ = corr(*x*_*g*_1__, *x*_*g*_2__) is the Pearson correlation coefficient between the pair of genes *p* = (*g*_1_, *g*_2_). The IC score for gene *k*, defined as *I*(*s* ^1^, *s* ^2^) = corr(*ρ*_*p*_^*s*^1^^, *ρ*_*p*_^*s*^2^^), *p* = (*g*_*k*_, *g*_*i*_), *i* ≠ *k*, quantifies the coherence between the studies *s* ^1^ and *s* ^2^. In this study, an IC score was calculated for each gene and the genes with IC scores smaller than the median of those IC scores were filtered out.

### 2.5. Statistical Metrics

As in the previous study [5], we used four metrics, Belief Confusion Metric (BCM), Area Under the Precision-Recall Curve (AUPR), Generalized Brier Score (GBS), and misclassified error rate, to evaluate the performance of a classifier. The references therein described those metrics in detail. Briefly, they all range from 0 to 1. For the first two metrics, the closer to 1 the better classifier, whereas the direction is opposite for the last two metrics.

### 2.6. Statistical Language and Packages

Statistical analysis was carried out in the R language version 3.1 (https://www.r-project.org/), and R codes for SAMGSR were downloaded from Dr. Yasui's homepage (http://www.ualberta.ca/~yyasui/homepage.html).

## 3. Results

### 3.1. Application of SAMGSR

**Figure 1:**
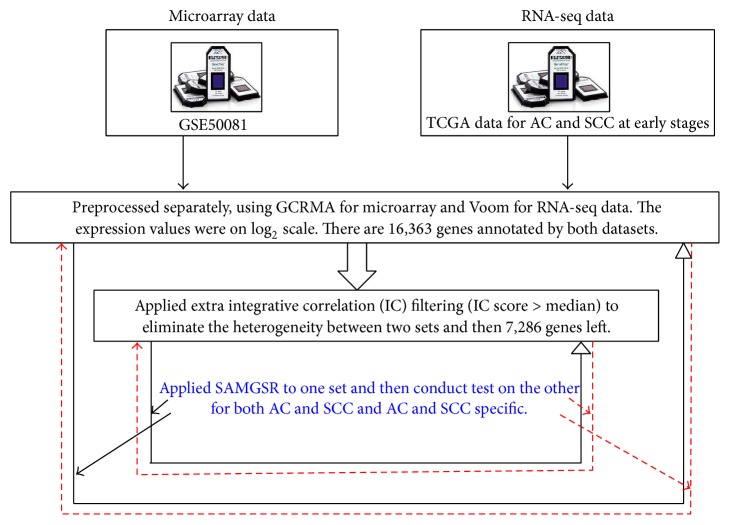
Study schema. Graphical illustration on the applications of SAMGSR to the stage segmentations of early-stage NSCLC.

We analyzed the NSCLC data using SAMGSR. We first used the microarray data (GSE50081) as the training set and the RNA-seq data as the test set. Then, we swapped them and applied SAMGSR again.  Figure 1 illustrates how the analyses were carried out, and Table 1 presents the calculated performance statistics.

In Table 1, several patterns are apparent. First, IC filtering tends to improve the performance of resulting classifiers by eliminating those genes with inconsistent expression patterns. For example, for the stage segmentation when trained on the RNA-seq data, AUPR increases from 0.529 before IC filtering to 0.623 after IC filtering while BCM increases from 0.523 to 0.569. Additionally, the model parsimony after IC filtering improves in most of these segmentations. For instance, for the same stage segmentation, the size of the final model decreases from 52 to 24 after implementing IC filtering. This observation indicates that prefiltering before the implementation of a more complicated algorithm facilitates the process of feature selection, by the means of screening those genes more likely to be irrelevant out.

Second, to evaluate a classifier's performance on the basis of several statistical metrics, we have demonstrated that different metrics may focus on different aspects of a classifier, and the superiority of an algorithm drawn based on only one or two statistics might not be solid. Additionally, it is observed that no algorithm can outperform the other methods in terms of all performance metrics. Instead, one algorithm is more likely to be superior in some metrics but inferior in others. Thus, a more thorough evaluation using different performance statistics might help to characterize an algorithm better and is highly recommended.

Third, all stage segmentations for the SCC subtype at least perform comparable to those for the AC subtype but the number of genes being selected in the SCC segmentations is less, which deviates from the results from our previous study [5]. Nevertheless, this is in accordance with the fact that AC is divided into more molecular subtypes and is more heterogeneous than SCC [3]. We note that there exist two major differences between this study and the previous study. In that study, a regularization method called threshold gradient descent regularization (TGDR) [27] was used to carry out feature selection, and a different data set whose ratio of stage II samples to stage I samples is also away from one was chosen as the training set. Therefore, we conjecture that TGDR algorithm, which might be very sensitive to the imbalance of sample size between two groups, and the different characteristics in two study populations contribute to this inconsistence. Further study is warranted.

Finally, stage segmentations for either AC or SCC specific have better performance compared to those without stratifying on the histology subtypes. Although this pattern does not hold uniformly for all comparisons, it still suggests that diagnostic gene signatures for these two subtypes might differ.

### 3.2. Comparison with Other Algorithms

Here, we compared SAMGSR with several novel feature selection algorithms to show that the reduction step of SAMGSR can be considered as a process of feature selection. The feature selection algorithms under consideration include least absolute shrinkage and selection operator (LASSO) [28], penalized SVM [29], moderated *t*-test to identify differentially expressed genes (DEGs) [21], and Radviz [30].

The first three algorithms have been widely used for variable selection. LASSO was implemented using the glmnet package [31] in R. The tuning parameter *λ* in LASSO controls the amount of regularization. In general, a lower *λ* value leads to less regularization, corresponding to an increased number of nonzero coefficients while a higher *λ* value corresponds to a sparser model. With 100 different *λ* values, 5-fold cross-validations were performed. The *λ* value that minimized the classification error was chosen.

The penalized SVM algorithm was implemented using R penalized SVM package [29]. In penalized SVM, we chose to use a Smoothly Clipped Absolute Deviation (SCAD) penalty [32] which has two tuning parameters *α* and *λ*. *α* was set at its default value of 3.7. Then, for the grid of 2^−8^, 2^−7^, 2^−6^,… and, 2^14^, *λ* was optimized using 5-fold cross-validations (CV), that is, its optimal value corresponding to the one with the smallest 5-fold CV classification error.

The moderated *t*-tests were implemented using R limma package [33]. The Benjamini and Hochberg procedure [34] was used to adjust for multiple comparisons. In this study, we consider the significance level in a moderated *t*-test as a tuning parameter. Namely, for the grid of 0.01, 0.05, 0.1, 0.15,… and, 0.3, the cutoff of adjusted *p* value was set as the one with the smallest 5-fold CV error.

Radviz, a visualization tool, may be also utilized to carry out feature selection as shown by us [5]. In that study, we had also described how Radviz selects relevant genes. Briefly, setting the maximum number of features under consideration from 3 to 10, the VizRank approach [35] was used to search for a combination of genes with the largest degree of class separation. Furthermore, since for the last two methods the classifiers are not automatically produced along with the process of feature selection, we fitted SVM models to estimate corresponding coefficients before the selected genes.

The performance statistics are presented in Table 2. Overall, SAMGSR performs comparable to these four feature selection algorithms. For example, for the AC-specific stage segmentation SAMGSR has a BCM score of 0.609 and an AUPR score of 0.63, ranking at the first and the second places, respectively, among the five methods.

### 3.3. Biological Interpretation of SAMGSR Results

**Figure 2:**
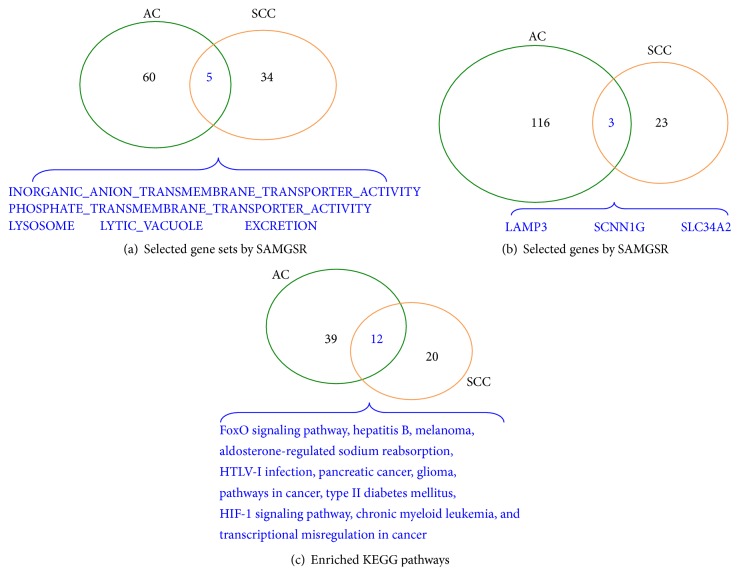
Venn diagrams show how the selected gene sets and genes for SCC and AC stage segmentations overlap. (a) On the level of gene sets selected by SAMGS. (b) On the level of genes selected by SAMGSR. (c) On the level of enriched KEGG pathways. There are 5 overlapped gene sets, 3 overlapped genes, and 12 overlapped KEGG pathways, respectively.

Upon the signatures obtained from the microarray data, we further explored on the biological meaning of those selected gene sets and genes. We first focused on those selected gene sets by SAMGS. Subsequently, we moved to those individual genes selected by the reduction step. Roughly 90% of genes involved in those selected gene sets were screened out in this step; some of the selected gene sets by SAMGS might consequently lose their significance. Therefore, we returned to the gene set level again by carrying out KEGG pathway enrichment analysis upon the 119 genes and 26 genes for AC and SCC, respectively. The pathway enrichment analysis was conducted using the STRING software (http://string-db.org/). Venn diagrams in Figure 2 illustrate how those gene sets and individual genes identified by SAMGSR and those enriched KEGG pathways for AC and SCC overlap.

On all levels, the size of overlaps between SCC-specific and AC-specific stage segmentations is small. This justifies partially that SCC and AC are distinct diseases, indeed. Nevertheless, the proportion of overlaps on either gene set or enriched KEGG pathway level is several times larger than that on individual gene level, which is in accordance with the fact that the consistency/stability of selected gene sets/pathways from different studies is better than that of selected individual genes [36]. To conclude, we suggest that stratified analysis on each specific subtype should be conducted. Alternatively, one may resort to more complicated statistical methods targeting at subtype specific genes, for example, [20, 37] when constructing diagnostic or prognostic gene signatures of these two subtypes.

We searched on the GeneCards database (http://www.genecards.org/) and found that, among 26 genes identified by SAMGSR for SCC subtype, there are 8 genes directly related to SCC, 4 to NSCLC, and 10 to LC, respectively. The overlapped genes that are directly related to NSCLC, LC, and SCC include* IVL*,* TGM1*,* NEU1*, and* SFN*. Furthermore, the remaining genes are all indirectly related to either SCC or NSCLC/LC. Since we compare stage II with stage I of SCC, we remark that these genes are not only differentially expressed between SCC cases and controls, but also differ quantitatively between these two stages of SCC. Those four common genes with literature-supported association with SCC and NSCLC/LC might be the “driving” genes capable of distinguishing SCC-I from SCC-II. Their potential as biomarkers deserves further investigation.

Similarly, among the selected 119 genes for AC subtype, there are 43 genes directly associated with AC, 28 with NSCLC, and 42 with LC, respectively. And there are 22 overlapped genes including* POLB*,* FGFR4*,* TGFBR2*,* HPGD*,* STC1*,* SLC3A2*,* AGER*,* GDF10*,* POLI*,* NTRK2*,* PTGER4*,* PIK3R1*,* EDN1*,* IL6R*,* AQP4*,* SFTPD*,* ID1*,* TIMP1*,* MMP7*,* IL12RB2*,* ERBB3*, and* SLC7A5*. Except those directly related genes, the rest of genes are indicated as indirectly related ones. Thus, the genes selected by SAMGSR have some biologically meaningful implications. Nevertheless, we remark that those resulting signatures by SAMGSR cannot be used in the clinical setting right away since the pathway databases are incomplete and subject to changes. Further investigations are demanded.

## 4. Conclusions and Discussion

When SAMGSR was applied to identify genes capable of discriminating AC and SCC apart, it selected more than one hundred genes besides* KRT5* (data not shown). In contrast, Ben-Hamo et al. [38] used only* KRT5* to separate AC and SCC apart with an accuracy of around 85% in the sbv Improver challenge [39]. This indicates that the final models by SAMGSR might include many irrelevant genes. Since SAMGSR may be classified as a filter-typed feature selection algorithm, it inherits the inferiority of a filter model in terms of model parsimony. Special care to eliminate the false positives by SAMGSR is needed.

Besides its inferiority in terms of model parsimony, the SAMGSR algorithm has two more drawbacks. First, if the true markers are not involved in any annotated gene sets, it is impossible for SAMGSR to identify them. Furthermore, the number of gene sets inside which a gene is contained has impact on its chance of being selected. If the true markers are just involved in few gene sets, SAMGSR has high likelihood to miss them given that the gene sets containing these markers might be even ruled out by SAMGS at the first place. Second, the SAMGSR algorithm does not take the pathway topology knowledge into consideration. SAMGSR only assumes that genes inside the “core” subsets function together to produce influence on biological processes and weighs all genes in those “core” subsets equally. Currently, we are working on an extension to SAMGSR, in which the genes with subtle changes but high connectivity with other genes are considered to be of more importance and thus endowed with larger weights. The weighted version of SAMGSR will be present in another paper.

To the best of our knowledge, however, no research except our previous work [11] has explored the feature selection trait possessed by some gene set analysis algorithms and adopted them directly for feature selection. Because the pathways in those canonical databases had been usually coined based on diseases under extensive investigation such as cancers, the multiple sclerosis dataset we used in the previous study might be less suitable to justify that SAMGSR is a feature selection algorithm. Here, the application of SAMGSR to NSCLC data provides more evidence on the fact that SAMGSR can conduct feature selection, given that SAMGSR performs comparable to several novel feature selection algorithms. Besides SAMGSR, we note that other gene set analysis methods such as [40] can be adopted directly or modified correspondingly to carry out feature selection. Therefore, the work here will boost the real-world applications of those gene set analysis methods and propel the development of pathway-based feature selection algorithms.

## Figures and Tables

**Table 1:** Performance of SAMGSR on NSCLC data for stage segmentations.

	Training set				Test set^*∗*^			
	Error (%)	GBS	BCM	AUPR	Error (%)	GBS	BCM	AUPR
(A) Trained on the microarray data (GSE50081)								
No IC filtering, on stage (115)	1.18	0.050	0.809	0.976	32	0.318	0.51	0.612
No IC filtering, for AC (83)	0	0.039	0.825	0.996	35.7	0.357	0.5	0.627
No IC filtering, for SCC (14)	7.14	0.082	0.758	0.957	43.6	0.308	0.511	0.513
With IC filtering, on stage (75)	5.92	0.067	0.784	0.964	36	0.344	0.56	0.535
With IC filtering, for AC (119)	0	0.043	0.810	0.996	42.9	0.350	0.609	0.630
With IC filtering, for SCC (26)	2.36	0.062	0.802	0.992	32.7	0.256	0.589	0.583

(B) Trained on the RNA-seq data								
No IC filtering, on stage (52)	0	0.028	0.871	0.997	30.8	0.270	0.523	0.529
No IC filtering, for AC (14)	11.43	0.087	0.779	0.961	58.4	0.454	0.533	0.536
No IC filtering, for SCC (28)	0	0.035	0.842	0.991	45.2	0.278	0.532	0.563
With IC filtering, on stage (24)	12.8	0.110	0.725	0.873	38.6	0.272	0.569	0.623
With IC filtering, for AC (31)	0	0.033	0.848	0.995	30.7	0.258	0.533	0.576
With IC filtering, for SCC (10)	9.09	0.101	0.712	0.905	33.3	0.279	0.556	0.641

Note: ^*∗*^the test set is RNA-seq data in part (A) and GSE50081 microarray data in part (B).

**Table 2:** Comparison of SAMGSR with other feature selection algorithms.

Method	Subtype	Training set				TCGA RNA-seq			
		Error (%)	GBS	BCM	AUPR	Error (%)	GBS	BCM	AUPR
SAMGSR + SVM	AC (119)	0	0.043	0.810	0.996	42.9	0.350	0.609	0.630
	SCC (26)	2.36	0.062	0.802	0.992	32.7	0.256	0.589	0.583

Lasso	AC (81)	0	1.14 × 10^−4^	0.990	0.996	35.7	0.357	0.5	0.624
	SCC (33)	0	<10^−4^	0.993	0.992	29.1	0.291	0.5	0.565

Penalized	AC (528)	0	0.003	0.951	0.996	37.1	0.318	0.524	0.615
SVM (SCAD)	SCC (63)	0	<10^−4^	0.999	0.959	27.3	0.273	0.531	0.654

DEGs + SVM	AC (145)	0	0.042	0.810	0.996	51.9	0.465	0.562	0.638
	SCC (46)	0	0.046	0.803	0.992	29.1	0.287	0.501	0.632

Radviz + SVM	AC (9)	22.83	0.166	0.559	0.734	37.1	0.363	0.493	0.541
	SCC (8)	4.76	0.076	0.774	0.934	30.9	0.293	0.493	0.536
