# Corrigendum to “Classification of Non-Small Cell Lung Cancer Using Significance Analysis of Microarray-Gene Set Reduction Algorithm”

**DOI:** 10.1155/2018/6031094

**Published:** 2018-03-20

**Authors:** Lei Zhang, Linlin Wang, Bochuan Du, Tianjiao Wang, Pu Tian, Suyan Tian

**Affiliations:** ^1^School of Life Science, Jilin University, 2699 Qianjin Street, Changchun, Jilin 130012, China; ^2^Department of Neurology, The Second Hospital of Jilin University, 218 Ziqiang Street, Changchun, Jilin 130041, China; ^3^Division of Clinical Epidemiology, The First Hospital of Jilin University, 71 Xinmin Street, Changchun, Jilin 130021, China; ^4^School of Mathematics, Jilin University, 2699 Qianjin Street, Changchun, Jilin 130012, China

In the article titled “Classification of Non-Small Cell Lung Cancer Using Significance Analysis of Microarray-Gene Set Reduction Algorithm” [1], an acknowledgment should be added as follows.

“The authors thank Dr. Howard Chang for English editing. This study was supported by the National Natural Science Foundation of China (31401123 and 31270758).”

Additionally, the email address of the second corresponding author should be corrected to “windytian@hotmail.com.”
